# Self-Ascribed Paranormal Ability: Reflexive Thematic Analysis

**DOI:** 10.3389/fpsyg.2022.845283

**Published:** 2022-04-12

**Authors:** Kenneth Graham Drinkwater, Neil Dagnall, Stephen Walsh, Lisa Sproson, Matthew Peverell, Andrew Denovan

**Affiliations:** ^1^Department of Psychology, Manchester Metropolitan University, Manchester, United Kingdom; ^2^Adelphi Values Ltd., Bollington, United Kingdom

**Keywords:** self-ascribed paranormal ability, reflexive thematic analysis, psychics, mediumship, lived experience

## Abstract

This study investigated personal perceptions (involvements) and comprehensions (interpretations) of self-ascribed paranormal abilities. Twelve participants with supposed supernatural powers took part in semi-structured interviews exploring the origin, phenomenology, and nature of their powers. Interview transcripts were analysed using reflexive thematic analysis (RTA), a qualitative method that identifies patterns within data. Four major themes expressed meanings and representations held by participants: Formative Influences (sub-themes: Gifted Family Members and Anomalous Occurrence), (Inter) Subjective Paranormal Experience (sub-themes: Transcendental/Mystic and Extra-Sensory Perception), Embodied Processes (sub-theme: Control), and Perception of Reality (two sub-themes: Self-Awareness and Fantastic/Surreal Perceptions). Consideration of themes identified an inextricable link between perception, interpretation, and belief in ability. Within narratives, interviewees outlined, contextualised, and established the validity of their powers. They drew upon supporting autobiographical evidence from their life histories and obfuscated and/or discounted conventional explanations. Generally, accounts reflected individual attempts to comprehend and justify the nature and experience of professed abilities. The authors discuss these processes and suggest ways to extend and develop ensuing research.

## Introduction

The present manuscript investigated personal perceptions (involvements) and comprehensions (interpretations) of self-ascribed paranormal abilities. Although, researchers have historically focused on supernatural “beliefs” and “experiences” rather than ability (e.g., Dagnall et al., 2016; Drinkwater et al., 2020), this topic is important for several reasons. Theoretically it is necessary since the psychology of ability is relatively under researched compared to belief and experience (Drinkwater et al., 2021a,c, 2022), Thus, investigation of self-ascribed capabilities extends conceptual understanding of paranormal attributions. Explicitly, it provides insights into complex interactions between credence and direct participation. Additionally, at a practical level, scholarly work is required because paranormal ascriptions are associated with both adaptive (i.e., a cognitive “defence” against acceptance of the uncertainty of life events) and maladaptive (i.e., psychopathology) functioning (Williams and Irwin, 1991), and concomitantly related to well-being (Göritz and Schumacher, 2000).

Commonly self-ascribed paranormal abilities include, but are not restricted to mediumship, psychic power(s), spiritualism, and fortune-telling (see, Drinkwater et al., 2021a,b). Mediumistic occurrences represent a type of transpersonal experience, where the participant’s sense of identity transcends corporeal boundaries, allowing “gifted” individuals to access information not normally considered obtainable (Daniels, 2005). Such material includes sensations from the deceased, and in some instances the use of spirit guides to mediate communications with the departed (Rock et al., 2008). Psychics claim ownership of one or more paranormal abilities (e.g., telepathy and precognition), which enables them to acquire data beyond the known limits of their normal senses. Spiritualism derives from the belief that it is possible to contact pneumas of the dead. Fortune-telling arises from the notion that “gifted” individuals can foresee the future. Although these basic delineations are not inclusive of all alleged supernatural powers, they provide crucial snapshots into the nature and types of supposed capabilities that have served as the focus for much preceding research. Supernatural in this context is used as a synonym for “paranormal,” which denotes to the acceptance of “a proposition that has not been empirically attested to the satisfaction of the scientific establishment but is generated within the non-scientific community and extensively endorsed by people who might normally be expected by their society to be capable of rational thought and reality testing” (Irwin, 2009, pp. 16, 17).

Most academic work examining self-ascribed paranormal abilities has been quantitative, concentrating on measurable outcomes and the identification of factors predicting self-ascription of professed paranormal abilities (e.g., astrology, McGrew and McFall, 1990; mediumship, O’Keeffe and Wiseman, 2005; Beischel, 2007; psychic powers, Parra and Argibay, 2009; spiritualism, Roxburgh and Roe, 2011). This positivist approach is integral to determining whether paranormal abilities exist and recognising psychological variables that influence personal perceptions of ability. However, it fails to fully inform understanding of individual involvement and meaning making. Specifically, how individual narratives inform construction of personal (*their*) supernatural realities (Ashworth, 2008). This is not an issue with positivism but rather arises from constrained research breadth (i.e., too few studies, focus on specific abilities, and failure to examine sufficient populations). The consequence is a lack of refined research instruments and limited data. Accordingly, qualitative/phenomenological/lived-experience can be seen as a complementary transition, which facilitates a fuller understanding of self-professed paranormal ability.

Acknowledging this, investigators have undertaken qualitative studies exploring the nature of paranormal ability and experience. These have evolved from the seminal phenomenological work of pioneering researchers such as Eugène Osty and Gerda Walther. Osty (1913) conducted comparative studies of psychics, mediums, and fortune-tellers. Then later, as the director of the Institut Métapsychique International, Osty (1926) invited psychics to describe their own abilities. Walther was a student of Husserl, who applied his phenomenology approach to the study of parapsychology. Illustratively, Walther produced “Phenomenology of Mysticism” based on personal mystical experiences. The content explored how spiritual beings are distinct from mere psychical beings and distinguished mystical lived-experiences and their objects from other forms of experience (Parker, 2018).

Much contemporary research has emphasised occurrence and demonstration rather than dispositional ownership. For instance, Roxburgh and Roe (2013) conducted semi-structured interviews with spiritualist mediums investigating their lived experience. This is relevant to the present manuscript as spiritualism is a key concept within paranormal research. Explicitly, in this instance it denotes the belief that “gifted” individuals mediate communication between spirits of the dead and living human beings. Using interpretative phenomenological analysis (IPA), three themes were identified: purpose, explanatory systems, and spirit guides as transcendental beings versus aspects of self. This analysis provided insights into the denotation mediums assign to their perceived abilities.

Other studies have considered mediums alongside psychics. Illustratively, Rock et al. (2009) asked six certified research mediums to describe their subjective experiences during discarnate communication and psychic readings for the living. Thematic analysis identified multiple constituent themes for both mediums (i.e., verificatory “sign” of contact with a discarnate) and psychics (i.e., the experience of “just knowing”). These were then located within the interpretive framework of Grof (1975) cartography of the transpersonal dimensions of the psyche, which describes the fundamental types of experience available to the typical person.

In another study, Beischel et al. (2017) investigated reported experiences of “secular” American mediums (those not associated with any formal religious organisation) during readings involving communication with the dead and psychic readings for the living. Respondents completed open-ended, online survey items. Qualitative analysis, in the form of content analysis, identified three overarching themes for mediumistic communication: preparation, communication triangulated, and experience of the communication. For psychic readings, four summative themes were identified: establishing the connection, experiencing the connection, content of the reading, and psychic information flowing from various sources.

Collectively, work such as these have enhanced academic appreciation of personal perceptions of paranormal ability by demonstrating that self-ascribed categorisations are associated with “particular” meanings and understandings. Despite this, further investigation is necessary since previous research has focused on only a narrow range of paranormal abilities and/or special groups. These are not representative of the array of powers considered by prevailing belief (see Dagnall et al., 2010) and experience (Dagnall et al., 2016) scales. Explicitly, those assessed featured in the two most prevalently used measures, the Australian Sheep-Goat Scale (Drinkwater et al., 2018) and Revised Paranormal Belief Scales (Drinkwater et al., 2017b).

While paranormal classifications are historically and conceptually useful, they are limited since individuals often profess multiple abilities, and therefore do not fit into neat categories. For these reasons, individuals often define their powers in terms of phenomenology, where judgments are guided by personal values and ethics. In this context, a frequent distinction is made between “sensitives” and “intuitives.” Despite lacking scholarly operationalisation, this categorisation is important since it signifies variations in self-attributions. Intuitives are conscious of events that have or could occur, whereas sensitives receive psychic information, which they interpret (Drinkwater et al., 2021a). Moreover, individuals regularly distinguish between extemporaneous (external/spontaneous) and measured (internal/controlled) capabilities (Drinkwater et al., 2013, 2017).

### Current Research

Noting these conceptual issues, the present manuscript focused on “general,” personal accounts of self-ascribed paranormal abilities. Reflexive thematic analysis (RTA) was used because it acknowledges the active role of the researcher in knowledge production and eschews positivistic ideas pertaining to data interpretation (Liu et al., 2020; Byrne, 2021).

## Materials and Methods

### Use of Reflexive Thematic Analysis

Reflexive thematic analysis (RTA) is an interpretivist paradigm, which places an emphasis on understanding the subjective experiences of individuals. Since the authors were investigating the “meanings” that participants created and attributed to their paranormal ability-related experience, RTA was an appropriate analytical approach. RTA was selected because it can identify patterns within a data set, while permitting conceptually informed interpretation of meaning. Since RTA is not restricted by methodological commitments, analysts are able to draw on the theoretical framework of their choosing to make sense of data (D’Souza et al., 2020).

Within the present study, the authors viewed self-processed paranormal ability as a “psychological” rather than parapsychological process, analysis was broadly informed by an attributional approach (Drinkwater et al., 2019; O’Keeffe et al., 2019; Laythe et al., 2021). This acknowledges how the social perceiver uses information to construct and rationalise deterministic inferences and explanations (see D’Souza et al., 2020). Central to this, is how participants collate and integrate information to produce causal judgements, which shape and structure ability-related experience, meanings, and assumptions (Braun and Clarke, 2013). From this perspective, RTA enables assessment of personal psychic abilities from a psychological, social, and cultural perspective rooted in social constructionism (Braun and Clarke, 2019). This is commensurate with Braun and Clarke (2020), who emphasise the need for researcher transparency regarding paradigmatic, epistemological, and ontological assumptions. Thus, use of RTA enabled detailed investigation of the impact and significance of self-ascribed paranormal abilities from the individual’s viewpoint (Murray and Wooffitt, 2010).

Moreover, RTA allowed thematic examination of the construction of meaning and experience through narrative. To generate rich accounts, the researchers used semi-structured interviews. These focused on how individuals surround personal practice (formal and/or informal) with explanation and considered the role of belief. By examining individual perspectives, interviews provided insights into the thoughts, perceptions, and feelings that characterise self-ascription of paranormal ability. Particularly, how individuals make sense of their powers in terms of the self, society, and world. Subsequently, this research cultivated novel elucidations for perceived abilities.

### Participants

Twelve (nine females and three males) individuals from the United Kingdom, United States, and Europe with self-ascribed paranormal abilities agreed to participate in interviews. They volunteered after taking part in self-report studies assessing paranormal features (i.e., beliefs, experiences and abilities) and individual differences in cognitive perceptual processes and executive function. Debriefs in these studies, invited participants with supposed abilities to contact the principal investigator via email if they were willing to discuss their powers in a further research project. Twenty-six individuals followed up this invitation. Eighteen provided content and progressed to interview. Prior to interview, participants signed a consent form (agreeing to take part and digital recording).

The principal investigator, using a function in Excel, randomly selected twelve interviewees (nine females and three males). Generally, this figure is regarded as appropriate for qualitative research (see Kuzel, 1992, 1999; Morse, 1994), Moreover, it permits data saturation (Braun and Clarke, 2006, 2019; Guest et al., 2006; Bragaru et al., 2013). Interviewee accounts encompassed a range of self-ascribed paranormal abilities (real names replaced with pseudonyms to protect identity; see Table 1). No interviewees refused to participate or wished to withdraw. All interviews occurred only once; there were no repeat interviews, and no field notes were taken.

**TABLE 1:** Participants and their professed abilities.

Transcript	Pseudonym	Age (years)	Preferred gender	Self-professed ability
Transcript 1	Jessica	21	Female	Sensitive
Transcript 2	Tony	28	Male	Psychic
Transcript 3	James	57	Male	Psychic/medium
Transcript 4	Suzanne	46	Female	Sensitive
Transcript 5	Craig	54	Male	Psychic
Transcript 6	Rebecca	59	Female	Medium
Transcript 7	Sarah	66	Female	Clairvoyant/remote viewer
Transcript 8	Louise	57	Female	Psychic
Transcript 9	Alison	47	Female	Tarot card, reader, divination, psychic
Transcript 10	Stephanie	45	Female	Psychic/medium
Transcript 11	Cathy	74	Female	Clairvoyant/spiritualist
Transcript 12	Elizabeth	54	Female	Spiritualist/medium

### Procedure

Semi-structured interviews were conducted between November 2020 to April 2021 by the lead author (KD). KD (53-year-old male) has a Ph.D. in Psychology and is a Senior Lecturer. He is an established researcher, with 15 years of experience, who is well published in the areas of quantitative and qualitative psychology and parapsychology. KD possesses a good knowledge of the paranormal, and although sceptical about the existence of supernatural powers is compassionate to those who believe and report experiences and abilities. Subsequently, he is non-judgmental, and appreciates the impact that paranormal beliefs/experiences have on individuals, and is keen to learn more about personal encounters.

Prior to commencement of interviews the researchers produced a semi-structured interview guide composed of questions pertaining to individual paranormal ability [e.g., (1) Please can you tell me if you are or have ever been a psychic/a medium/or a clairvoyant? If, yes then…What do you think being one of these means to you, and which one do you feel best describes/fits you? (2) If you have a psychic ability, can you explain how this works and what you do that involves your ability?]. These were piloted in advance with two of the researchers acting as naïve respondents.

Interviews lasted approximately 57 mins (between 36 and 94 mins) and were audio recorded. Supplementary field notes were also taken by the interviewer. The protocol included a general introduction and concluded with a debrief. Interviews took place over Skype or Zoom (telecommunication platforms that facilitate video telephony, conferencing, and voice calls) at times and dates agreed by both the lead author and individual participants. Interviews were transcribed verbatim and anonymised; to protect the identity of interviewees names were replaced with pseudonyms and personal identifying characteristics removed.

This virtual environment provided a safe and secure formal setting, where meetings could occur without interruption. Specifically, it served as a friendly, relaxed environment, where interviewees could freely outline their personal perceptions and experiences. Each interview began with respondents reading the participant information sheet and listening to a short brief about the nature of the research. The interviewer (KD) explained the nature of the study and outlined the procedure, which included actions for data storage (i.e., recording), anonymisation, and the transcription process. Prior to recording, interviewees were reminded of their right to terminate or withdrawn from the study at any time prior to the analysis phase and write up of the research study. These procedures established a shared understanding and empathy between researcher and participant. Although interviewees were offered the opportunity to view their final transcripts, none accepted this offer.

### Ethics Statement

Ethical approval was provided by the Manchester Metropolitan University, Faculty of Health, Psychology and Social Care Ethics Committee (October 2018; Project ID, 954). The study adhered to British Psychological Society guidelines and ethics (The British Psychological Society, 2021).

## Results

### Analytical Approach and Justification

Data analysis used RTA. RTA was selected because the present study focused on the unique and subjective experiences of individuals with self-ascribed possess paranormal abilities. By providing a framework for organising detailed data responses, RTA facilitated identification, analysis and reporting of patterns within the interview data (Braun and Clarke, 2006, 2013). Explicitly, RTA allowed interpretation of characteristics inherent within the research topic.

Moreover, RTA was located with the researchers’ social-constructionist epistemological position (Andrews, 2012; Willig, 2013) and provided a flexible process of analysing interview transcripts that enabled an organic development of themes (Braun and Clarke, 2006, 2013). This is consistent with the notion that RTA represents “the researcher’s reflective and thoughtful engagement with their data and their reflexive and thoughtful engagement with the analytic process” (Braun and Clarke, 2019, p. 594). Accordingly, RTA is the researcher’s interpretive analysis of data conducted at the intersection of the theoretical assumptions of the analysis, and the analytical skills/resources of the researcher (Braun and Clarke, 2019).

As such, there is a deductive, or theory driven, aspect to the analysis. However, there is no expectation that researchers will connect these criteria in the same manner. Thus, codes or themes produced are likely to vary (Byrne, 2021). Nonetheless, multiple coders are useful to check ideas and explore assumptions and/or interpretations. With multiple researchers RTA should be collaborative and reflexive (Byrne, 2021). That is, achieve richer interpretations of meaning, rather than consensus (i.e., a single, correct solution) (Braun and Clarke, 2013).

With RTA both coding and theme development are flexible, organic process that evolve during the analytical process (Braun and Clarke, 2019). Progression increases familiarity with data and may facilitate novel patterns of meaning. Themes are produced by organising codes around a relative core commonality, or “central organising concept,” that the researcher interprets from the data (Braun and Clarke, 2019). Consequently, there is also an inductive, or data driven, aspect to the analysis.

Reflexive thematic analysis is a purely qualitative approach, this definition pushes the use of RTA into exclusivity under appropriate qualitative paradigms (e.g., constructionism) (Braun and Clarke, 2019, 2020). As opposed to other forms of qualitative analysis such as content analysis (Vaismoradi et al., 2013), and even other forms of TA such as Boyatzis (1998) approach, RTA eschews positivistic notions of data interpretation. Thus, Braun and Clarke (2019) encourage the researcher to embrace reflexivity, subjectivity and creativity as assets in knowledge production. Notwithstanding the centrality of reflexivity, and the impossibility of extracting ourselves from these data, it is our intention that what we perceive to be the voices, and experiences, of our participants be foregrounded.

Reflexive thematic analysis employed the six-step process outlined by Braun and Clarke (2006, 2012, 2013, 2014, 2019): familiarisation with data, generate initial codes, generate themes, review potential themes, define, and name themes, and produce the report. This is not tied to any theoretical framework and is recursive in nature. In the present research, although the analysis cannot be described as a phenomenological analysis, it is fair to say that this is a reflexive thematic analysis with an eye to phenomenology – lived experience. Hence, while steps appear linear, researchers move backwards and forwards between them as themes are identified. For these reasons RTA is an evolving, time-consuming process (Byrne, 2021). New interpretations can arise that necessitate further iterations of previous phases. Due to this flexibility the six-step process is viewed as a set of guidelines rather than rules (Byrne, 2021).

Analysis involved all five members of the research team, who each independently examined transcripts. They then, via in-person meetings and email exchanges, discussed transcripts to agree on themes.

#### Themes

Following organic discussions and agreement within the research team, four themes, and sub-themes were discerned. For brevity, and consistent with other published academic papers, we selected pertinent examples, which were typical of “participants” (e.g., D’Souza et al., 2020; Campbell et al., 2021; Gibson et al., 2021).

(1) Formative Influences (two sub-themes: Gifted Family Members and Anomalous Occurrence). This theme was temporally focussed and identified how participants grounded their perceived ability in previous life history. (2) (Inter) Subjective paranormal experience (two sub-themes: Transcendental/Mystic and Extra-Sensory Perception/ESP). In this second theme, we noted how individuals described their sense of experience (i.e., the phenomenology) of their abilities. (3) Embodied Processes (sub-theme: Control). This theme overlapped with the previous one, but was distinct because it emphasised how, individuals experienced their paranormal powers. There was an applied/agentic dimension intrinsic to this theme, which contrasted with the passivity of the subjective paranormal experience theme. (4) Perception of Reality (two sub-themes: Self-Awareness and Fantastic/Surreal Perceptions). This theme identified the affect the expression of the ability had on the participants’ cognitions, perceptions, and sense of self.

The researchers noted *post hoc* that the agreed themes aligned with a commonly used heuristic for the lifeworld. Explicitly, temporality (the experience of time), inter-subjectivity (relationality, the experience of “other”), embodiment (channelling and control), and perception of reality. Participants linked the latter with space (spatiality).

In the context of quote selection, citations are illustrative; they represent both themes and the range of abilities expressed. Subsequently, not all participants’ comments are utilised.

##### Theme 1: Formative Influences (Two Sub-Themes: Gifted Family Members and Anomalous Occurrence)

This theme centred on formative influence(s). These were typically experienced during childhood and early adulthood. Interviewees outlined direct paranormal experiences and how they informed comprehension of their abilities. The grounding of perceptions in life histories indicated their profound personal importance. For example, James (psychic/medium) explained:

*“I’d have to say I’d more describe myself as a medium/clairvoyant because I’ve always had this ability of knowing there’s an afterlife*, *you know I’ve always felt it*, *knew it was there and being able to communicate it*, *with it*, *from being a very young child really*.”

Tony (psychic):

*“I had lots of P: experiences in my childhood of err*, *paranormal type nature*. *I mean*, *as a child*, *erm*, *growing up*, *I’d get a lot of erm*, *err*, *visions that would take place in the future*.”

This led him (Tony) to question what those encounters/experiences might be:

*“The experiences that I had and what might potentially happen*, *erm*, *upon and after death*. *I started to be more intrigued*, *I suppose have more conversations with certain family members around that sort of topic*. *And my mother*, *in her younger years had attended a spiritualist church and also seen different psychics and mediums for different things*.”

Interviewees outlined a wide range of self-professed paranormal abilities and methods of communication. ESP (the theoretical ability to perceive things by means other than the known senses) with the living, psychics connected with energy, and spiritualists channelled and communicated with the deceased. Within these, sense of time was central and added significance. Jessica (sensitive) explained that she had:

“*been gifted since I was a young child*,”

James (psychic/medium): *“I’ve always felt it*…. *from being a very young child really*.”

Craig’s (psychic) initial experiences began in early childhood with sightings of apparitions:

“… *when I was three and half*, *four*, *we moved into this house and that about a year or two later a ghost started to appear*. *We started seeing ghosts*.”

Rebecca (medium) reflected on her distant past when thinking about the experience of mediumistic processes:

*“I used to see orbs as a child when I was in bed and it was dark and there were these lights in my room*, *and it was dark and the scared bejesus out of me*, *but the phenomenon started*.”

###### Sub-Theme: Gifted Family Members

This sub-theme explored how interviewees’ belief in their abilities was influenced by family members. These acted as role models and normalised and legitimised paranormal powers. Interviewees described “gifted family members” possessing a range of supernatural powers (i.e., paranormal, sensitive, and magical). Jessica (sensitive) discussed how her relations were healers, mediums, psychics, witches etc. Suzanne (sensitive) described how she:

“*did have a great grandmother who was from the Ukraine*, *and she too was very sensitive and a bit magical*, *so she read tea leaves and she also read regular playing decks of cards*.”

For Sarah (clairvoyant/remote viewer), it was childhood experiences with her twin sister that convinced her that they possessed telepathic powers:

*“I have an identical twin sister and erm*, *and so*, *when we were really young*, *we would have*…*telepathic experiences*, *erm*, *but I would notice when I was a kid just like any*, *any time I was about to say something*, *I felt like I had to err*, *speak up really fast if I did want to say something or she’d say it first*.”

###### Sub-Theme: Anomalous Occurrence

The second sub-theme identified how early anomalous occurrences encouraged participants to reflect on the nature of their experiences and abilities. These led to both mundane and frightening occurrences that shaped both their perception and subsequent interpretation of encounters. Jessica (sensitive) explained:

*“I was around [pause] 13 when I first started to experience*, *so I got*, *I used to get night terrors*, *which is erm*, *pretty scary*, *but I think it was kind of*, *erm*, *err*, *isolated issue*.”

Craig (psychic) narrated a difficult experience, he had from his early school days. This made him question his personal reality in relation to other school children. Consequently, Craig assessed whether others share alike experiences, and interpretation them in similar ways:

*“And I’m thinking maybe you see the same ghost I do so you see this one ghost and it was different*, *so I didn’t understand that everybody has different experiences I thought just I did*, *but then maybe she’s seen the same one I’m seeing*.”

##### Theme 2: Subjective Paranormal Experiences, Intersubjectivity: (Two Sub-Themes: Transcendental/Mystic and Extra-Sensory Perception)

Interviewees reported myriad subjective paranormal experiences (SPEs). Within these it was the individual philosophy of sensed experience that informed paranormal beliefs and justified perceptions of power. Reported phenomena included sensed presence, spirits of the deceased, and contact with the dead. SPEs were discussed in terms of personal significance and shared elements of relationality and inter-subjectivity:

*“I’ve numerous sorts of experiences myself where err*, *presences*, *or spirits*, *or loved ones*, *or family that are deceased whatever you wish to call them*, *have*, *erm*, *you know*, *came to me and given me very specific messages in some cases not only for me but also for other people and obviously for clients as well”* (James, psychic/medium).

Rebecca (medium) similarly experienced others:

*“by the time I was 18 my father died*, *and it was really really sudden and I started seeing things at night*, *there would be things in my room I*’*ll be sleeping then I*’*d wake up and I called it the twilight stage between sleep and wake*…*things would often try and elicit reaction from me they*’*ll be throwing things at me*…*I*’*d wake up and find hats sitting on my pillow and think I was really startled*.”

###### Sub-Theme: Transcendental/Mystic Experience (Relationality)

This theme was intrinsically relational. Participants interacted and communicated with channelled forces, energies and sensed presences. These were often concomitant with vivid sensations and perceptual disturbance such as colours and feelings. Encounters also sometimes included the perception that something was moving through the participant. Elizabeth (spiritualist/medium) explained her experience in terms of her “tuning in” to perform mediumship, and the subsequent disruption related to her perceiving a spirit who was confused and disorientated:

*“You tune in as if you were doing mediumship*… *the guy that was causing the disruption in this instance umm it was because he had had dementia at the end and he hadn*’*t realised what was happened/he hadn*’*t really realised that he*’*d passed over*.”

Jessica (sensitive) outlined her earliest transcendental experiences of seeing a spirit where the energy and range of colours evoked a perception of specific resolution/purpose of the spirit:

*“I would experience blue especially when I was a kid*.*” “this is what I*’*m talking about colours*, *they communicate through colours and feelings and sensations*, *and so the colour of the lights of the car in the front when they broke red*, *the red completely took up my vision*.”…*”like a lighter and then the top of the flame is like that kind of energy*, *like you can see it in the air”*…*”it was almost that [pause] combined with [sic] like a slight light and then was coloured depending on the intentions of the spirit*. *And that would*, *it would normally be blue*.”

James (psychic/medium) expressed specific experiences from messages and communication with spirit. These involved feelings, receive, and hearing voices:

*“I’ve numerous sorts of experiences myself where err*, *presences*, *or spirits*, *or loved ones*, *or family that are deceased*, *whatever you wish to call them*, *have*, *erm*, *you know*, *came to me and given me very specific messages in some cases not only for me but also for other people and obviously for clients as well*.”

###### Sub-Theme: Extra-Sensory Perception

The extra-sensory perception (ESP) sub-theme related to specific telepathic connections. These varied as a function of the type and range of ESP-related powers explained. Tony (psychic): described how he connected telepathically and created the tuning event:

*“really it’s this sort of tuning my thoughts in*, *in*, *in a very subtle way in to connecting to telepathically to some other consciousness*…*it’s a bit like tuning in to a radio station really*. *People often say how do you create that connection and my answer’s well I don’t feel I create it*, *it’s always there*, *I’m just sort of tuning in to it*.”

Stephanie (psychic/medium) encountered mechanical abnormalities and items teleporting:

*“Things being moved*. *Things being moved to umm a different place in my house and I*’*m living alone*, *and I did not move them*, *and my cat could not have moved them or we*’*re sitting together*…*the lights going on*…*my nephew died first and then my father died*. *My TVs started to turn on*, *on their own*, *which was driving me crazy*.”

##### Theme 3: Embodied Processes (Sub-Theme: Control)

Although there were variations in the way in which processes were experienced (i.e., meditation, channelling, creating, and tuning in), interviewees generally agreed on their purpose and outcome. Particularly, that their paranormal powers provided a sense of control. James (psychic/medium), Tony (psychic), and Rebecca (medium) for example, who are specific practitioners, expressed a sense of importance, along with a belief that they controlled the mediation and channelling process. Additionally, juxtaposing their professional psychic careers, alongside regular clients’ forums and events in order to communicate, provided dialogue, and generated informal discussion, which enabled them to explore the nature of paranormal abilities.

For those who were not practitioners (e.g., Suzanne, sensitive; Cathy, clairvoyant/spiritualist; Louise, psychic; and Stephanie, psychic/medium), embodied processes and a sense of control were also important. Their abilities were defined by the ability to directly regulate incoming information. Suzanne (sensitive) presented the procedure as a constant:

“*I always feel like there is an on switch and it’s kind of always on*.” Moreover, she describes the source that flows through her.

*“I used to be a professional ballet dancer*, *and this is how I like to explain it to people*, *so if you have a certain body type you know*, *anybody can dance right*… *And that’s how I see the skill that I have*, *and I feel like everybody has senses obviously I feel it’s on a spectrum*.”

Jessica (sensitive) expressed her thoughts about the control she has, and how she employs meditation to regulate the communication and messages received.

*“Meditation is so key to controlling*, *erm*, *your ability and gifts*. *Erm*, *so*, *I come into like a state of meditation and then I kind of*, *err*, *I communicate with the spirit and say*, *I’m gonna open my channel*, *please can my loved ones and my guise help me through*.”

Some embodied processes were, however, difficult to explain. These were experienced as feelings and streams of information.

*“I don*’*t really think it comes from outside of me*, *whether it*’*s coming through me*, *I don*’*t/I*’*m not sure*, *but I know if I just/if I listen*, *like I*’*m listening*, *but I*’*m not listening to any voices outside*, *I*’*m just listening/it*’*s more like a feeling*. *And then the information just comes out”* Jessica *(sensitive)*.

Louise (psychic) explained the process of communication, where she tried to make sense of her ability whilst receiving information from within.

*“For instance*, *when I*’*m umm/when I*’*m communicating with someone who is departed*, *I don*’*t visually see them*, *I get an impression of you know what colour hair they had*, *their stature*, *their personality*, *that kind of stuff*, *like my grandmother came through one time*, *umm*, *and I didn*’*t know her*, *she had died probably 6 or 7 years prior to my birth*, *she died/I think she was only 40*, *and so stuff like that*, *yes*, *when I see/I don*’*t visually see them*, *I just get impressions”* (Louise, psychic).

Some interviewees had professional careers within the psychic world, where they conducted individual or groups sessions with clients. These often involved examining messages from deceased loved ones and family members (e.g., James, psychic/medium; and Tony, psychic). Other interviewees “merely” classed themselves as being either psychic or preferred the term sensitive (Suzanne, sensitive; Alison, tarot card reader/divination/psychic). The self-ascribed terms often crossed over between interviewees. Despite this, the range of abilities were wide ranging and diverse.

*“I*’*ve numerous sort of experiences myself where err*, *presences*, *or spirits*, *or loved ones*, *or family that are deceased whatever you wish to call them*, *have*, *erm*, *you know*, *came to me and given me very specific messages in some cases not only for me but also for other people and obviously for clients as well”* (James, psychic/medium).

###### Sub-Theme: Control

This sub-theme outlined being in control of the self-professed ability. Explicitly, that control was an integral part of the power that required moderation and management. James (medium/clairvoyant) discussing the process of mediumship:

*“have to control sometimes*, *is that emotion as I call it*, *because you can get somebody coming through quite emotional you know especially if you’ve just passed*, *or their loved ones there who wants to communicate so badly*, *and you’ve got to control that*…”

Jessica (sensitive) described acting as a sensitive:

*“yeah*, *it’s like control through meditation*…*it’s all about the power you have within yourself*. *You’re the stronger person as the living person*, *you’re*, *you are the one who has the say of whether like you wanna be in that or not*. *Erm you are the control*. *An aura of protection’*…*sometimes I can’t even control it*. *Like*, *especially when a spirit is*… *has a very urgent message*.”… *“I used to see spirits walking around the room because my channel was open constantly*. *Because*. *I didn’t know how to control it*, *but now I know how to control it*.”

##### Theme 4: Perception of Reality (Spatiality) (Sub-Themes: Self-Awareness; Fantastic/Surreal Perceptions)

This final theme revealed several encounters that participants had during visits. In this context, this theme explored self-awareness and lived space. Particularly, it was the countenance of abilities, and what impact these had on the participants’ thoughts, insights, and self-image. For example, Rebecca (medium) struggled with making sense of her reality given what she had witnessed/encountered. This was also apparent within transcripts where interviewees outlined distortions (in the sense that the world appeared to have changed visually in unusual ways), heightened emotions, and metaphysical occurrences. For instance, Rebecca (medium):

“…*It appeared in the corner of this room*, *image and then disappeared through the corner of the window and we were on the second or third floor and there*’*s no way to get out of the window and it was winter*, *so in the winter was closed and all that so at that point I started wondering if there*’*s some truth to what I was seeing if other people were seeing that too*.”

James (psychic/medium) explained how his encounters involved a meditation technique that involved telepathic connections and extending boundaries. This perception of truth, as it was experienced, helped to elucidate personal perceptions. Additionally, a connection within his own mind enabled him to make sense of both his conscious and subconscious thoughts from the world around him.

*“it’s when I go into my own self in a way*, *through the meditation*, *erm*, *and then essentially what I’m doing with the meditation is like I’m going into myself*, *quietening my own mind down and then I’m sort of stretching my consciousness out and I’m sort of then reaching out in a way in my mind to connecting to some other consciousness in that*, *in that strong as way as possible telepathically*.”

###### Sub-Theme: Self-Awareness; Fantastic/Surreal Perceptions

This final sub-theme illustrates how the experience of psychic/mediums/sensitives is framed in the lived experience of space. In both of the following quotes, there is a spatial immanency that is intrinsically entwined with the experience of space and proximity. Proximity to the point of centrality.

*“this anonymous voice that all of a sudden speaks out in your mind*…*it can be difficult to differentiate between what is maybe your own thoughts coming in*, *erm*, *a thought maybe on your part*, *maybe something from your subconsciousness”* Tony (psychic).

*“I’m going into an intentional state because I’m usually going into a mediative state first*.”… *“But sometimes it’s the physical like*, *I’ll just start to look at someone and I’ll see they’re only wearing one sock*… *There are physical things that come up and sometimes that seems to be a big challenge for all erm*, *people doing this*, *is*, *erm*, *knowing the difference between the symbols*, *is it symbolic or is it literal*, *others where it’s vaguer and you never quite understand what*, *you never get to the meaning of it”* (Sarah, clairvoyant/remote viewer).

## Discussion

The research set out herein investigated the experience, and interpretation, of self-ascribed paranormal abilities. Intrinsically, data and analysis provided insights into complex interactions between what individuals believe, their experiences, and their behaviour (abilities). At a practical level, this type of scholarly work, and these types of findings, are necessary, since paranormal ascriptions are associated with both adaptive (i.e., a cognitive “defence” against acceptance of the uncertainty of life events) and maladaptive (i.e., psychopathology) functioning (Williams and Irwin, 1991). Consequently, they influence well-being (Göritz and Schumacher, 2000).

Paranormal beliefs are intimately linked with culture (Ayar et al., 2022). Mind, is presented by Damasio (2018) as a cultural product, enabled by subjectivity and integrated experience. Crucially, Damasio (2018, p. 102) argues that “no satisfactory account of the human cultural mind is possible without factoring in affect.” Moreover, Damasio (2018, p. 104) proposes that feelings, affect, the “experiences of life based on multidimensional representations of configurations of the life process” are fundamental to being. In the proper sense of the term. To understand human being-ness, we must attend to the experience of mental life (Damasio, 2018, p. 100).

Relatedly, Ashworth (2015) outlines the need for qualitative researchers to attend to the “lived through” experiences of their participants’ life worlds. To this end, Ashworth proposes that everyone’s lifeworld can be understood in terms of fractions. Lifeworld fractions include the experience of temporality (time), inter-subjectivity (relationality), embodiment, and spatiality (the lived experience of space). It was only following the initial drafting of this present manuscript that we, the researchers, noted the fit between these lifeworld fractions and the themes identified during analysis.

Linking the reported experiences of our participants to the epistemological underpinnings of the present report: Our constructionist frame, as set out in the introduction, is deductive. Within this constructionist frame, we, the authors, set out to identify how participants construed their paranormal experiences and abilities. Inductively, we noted that the identified themes “meshed” with components of a commonly used heuristic for the lifeworld. As such, themes incorporated bottom-up and top-down considerations, or aspects. A key findings was that the experience of paranormal perceptions and abilities is central to our participant’s lived experiences, and essential to their life-worlds. The evidence presented in this report supports the idea that experience of paranormal perceptions and abilities sit easily within a heuristic, phenomenological, framework that applies to life-worlds generally. The experience of time, space, embodiment and inter-subjectivity is at the heart of what it is for a human being to “be,” and demonstrably fundamental to the experience of paranormal abilities.

Drawing on Damasio (1999), within interviews there was a distinction between “feeling,” the private, mental experience of emotion, and emotion the collection of responses that are openly observable. Thus, bodily responses are intangible from emotional and autobiographical perception. This is consistent with description of abilities were accompanied by valanced cognitions and affective responses. Furthermore, much of the narrative reflects personal attempts to make sense of feelings, perceptions and cognitions. This focus on feelings, the centrality of lived experience, has the practical effect of linking sense making with meaning and cognition (Smith et al., 2022). This demonstrates that the inherent theoretical flexibility (Braun and Clarke, 2012) of RTA “fits” well with a phenomenologically oriented thematic analysis. Indeed, one of the major theoretical assumptions underlying the present research (Braun and Clarke, 2019) was that phenomenology, lived experience, is the bedrock of self-ascribed paranormal ability.

To understand more about how individuals regard and experience their professed capabilities the authors explored whether self-designated capabilities shared mutual themes and patterns (see Drinkwater et al., 2013). To date no published study has investigated this question from the perspective of general abilities. Instead, previous work has focused on specific powers and/or self-identifying “gifted” groups. Although this has produced important conceptual insights into the psychology of self-ascribed paranormal abilities, it is limited to the extent that it is unclear whether the phenomenology of abilities such as mediumship resonates with other frequently reported facilities (extra-sensory perception, fortune telling, etc.). This was an important question to address because self-ascribed paranormal abilities do not typically conform to stereotypical domains and/or participants profess multiple abilities.

Consideration of general ability, like analysis of subjective paranormal experiences (see Drinkwater et al., 2017a), produced important insights into the personal perception of ability. Analysis indicated that self-ascription is a complex and sophisticated process. Commensurate with this, interviewees narrated rich and detailed accounts that made sense of their declared capabilities. Explicitly, they contextualised, rationalised, and provided evidence to support their claims. Thus, narratives contained a range of rich material referencing confirmatory background, processes (cognitive and affective), and phenomenology.

In this context, thematic analysis of interview transcripts produced a range of autobiographical references that delineated inextricable associations between life histories and possession of paranormal ability. These coalesced into meaningful and intelligible themes. Conceptually themes embodied connections between ability, experience, and belief. Psychologically, this suggested that self-ascription of ability, like experiences is explicable via the perception of an event/occurrence and the attribution (designation) of causation to paranormal powers or entities (Irwin et al., 2013; Lange et al., 2019).

Central to these processes is belief, which provides a framework for making sense of anomalous occurrences. Regarding ability, interpretation reciprocally reinforces supernatural credence and lived experiences. This view is commensurate with quantitative studies that report strong positive relationships between these distinct constructs. In the case of abilities this typically manifests as misattribution of external events to internal, dispositional paranormal forces. With experiences the ascription is general to external, supernatural agency (Dagnall et al., 2020).

Narratives demonstrated conviction and faith in the cogency of abilities. Interviews progressed through a series of typical phases. Initially, individuals delineated their abilities by drawing on background influences and then progressed to providing anecdotal, subjective supporting evidence. For instance, Alison (tarot card reader/divination/psychic) outlined stories about her grandfather who she felt influenced her:

*“My grandfather he died/I was in my early 20s when he died*. *He was quite a prankster and so in my house I have pictures all over the walls*. *Every once in a while*, *you know*, *there would be like straight picture*, *straight picture*, *a little askew*, *straight picture*, *straight picture and I think it was my grandfather because he would be the kind you know if you wanted things perfect he would just put a little bit off just to you know just agitate you and he*’*s done the pictures that you know you can*’*t even get to*.”

This was also true of Elizabeth (spiritualist/medium) who spoke about *“Powerful locations*.”

*“There*’*s also a tradition of following older spiritual practices*. *If you go to Wellington in Somerset*, *you can look along/walk along the streets and some of them are literally/there*’*s witch sort of logos in the window*, *followed by a gap*, *followed by a Born-Again-Christian*…*so it*’*s like that end of the woods is very much there*’*s a lot more sort of pagan stuff goes on as well*.”

Consistent with Drinkwater et al. (2017a), interviewees established and legitimised the authenticity of their accounts. As part of this process, they indicated that they were sharing important personal details. This conveyed sincerity in belief of ability, provided supporting evidence, and designated that interviewee had considered and discounted, mundane alternative explanations. Researchers have previously reported similar narrative devices in accounts of paranormal experience (Schmied-Knittel and Schetsche, 2005).

### Limitations

Though this manuscript followed recommended principles of best practice for qualitative research, issues pertaining to quality must still be considered. This study used the six-step thematic analysis approach of Braun and Clarke (2014). The approach to thematic analysis advocated by Braun and Clarke recognises the importance of quality in qualitative research. However, because the analysis is understood as something created by the research team, at the point where data, research experience, and academic knowledge meet, the focus on quality in the present manuscript pertains to rigor, a systematic approach, and thoroughness. As opposed to positivist concepts such as validity and reliability that do not mesh with the critical epistemology of the research presented herein (Terry et al., 2017).

Consistent with the guidelines of Guest et al. (2006), this study employed an adequate sample. Guest et al. (2006), following iterative assessment of coding, concluded that a sample size of 12 is frequently acceptable for interview studies exploring common experiences in a relatively homogeneous sample. Although, incidence of ability is under researched and therefore difficult to ascertain, recent research by Drinkwater et al. (2021a,b, c, 2022) designated that a significant percentage of respondents admit self-ascribed paranormal abilities. In the case of the Drinkwater et al. (2021a,c) studies, the sample sizes were relatively large (*n* = 917 and *n* = 499, respectively) and indicated that professed ability was relatively common. This figure is difficult to quantify because of the paucity of empirical work using general samples. However, Drinkwater et al. (2021a) observed that approximately 44% of respondents claimed some form of ability.

There were, however, limitations in sampling. Interviewees comprised respondents who followed up invitations from self-report studies to volunteer participation. In this context, they were self-selecting and motivated to discuss and outline their professed supernatural powers. Accordingly, the interviewees were likely to have strong conventions about the validity of their powers. Additionally, the number of males volunteering was significantly lower than females (approximately 25% were male). Although, this gender imbalance was typically of psychological studies of paranormal constructs (see Irwin et al., 2013; Dagnall et al., 2015; Denovan et al., 2018), there remains the possibility that themes were influenced by gender.

Regarding epistemically unwarranted beliefs, research has failed to report consistent gender differences (Lobato et al., 2014). However, differences in emotional expressiveness may have indirectly affected interviewee responses. These are evidence to support this supposition from work on death anxiety, where women scored higher than men on affectively oriented responses (Dattel and Neimeyer, 1990). Noting this, subsequent studies should consider whether accounts vary in content and focus as a function of gender.

Although, interviewees were self-selecting there was no reason to believe that these were atypical to respondents who typically report supernatural facilities. Clearly, this is something that subsequent research should investigate further since willingness to discuss abilities may be related to level of self-ascription. Hence, interviews could represent individuals who are most confident in their facilities and therefore extreme in terms of personal experience. The sample of interviewees were also representative of core abilities.

The alleged powers (i.e., sensitive, psychic, mediumship, clairvoyance, remote viewing, tarot reading, and spiritualism) corresponded closely with frequently reported paranormal experiences (see Dagnall et al., 2016), and aligned with core receptive elements of belief in the paranormal (Drinkwater et al., 2018). Despite these positive indicators, ensuing work should explore themes using further samples of self-ascribing individuals because data saturation is dependent on several factors such as the research question, design, sample, and context (Guest et al., 2006). This is important as themes derived from small samples are subject to great variability. Thus, an advantage of larger samples is they allow more typical accounts to be identified, which is useful to the determination of superordinate themes. Moreover, the presence of greater narrative content is likely to substantiate sub-themes (Roberson and Perry, 2021). These factors in combination will strengthen the conclusions about the nature of self-ascribed paranormal abilities.

Despite the identification of common themes, accounts were idiosyncratic and personal. This reflected variations in individual perception and interpretation. Thus, apparently comparable phenomena seem to have been experienced differently. For example, respondents claiming the ability to contact the deceased were involved in a variety of processes and practices. Additionally, profound formative influences impacted upon interviewees in myriad ways. Interpretative phenomenological analysis (IPA) is one method particularly well suited to further exploring these idiosyncrasies (see Wilde and Murray, 2010; Drinkwater et al., 2013), the lived experiences that were noted in the present research, because: One, IPA is committed, in an idiographic manner, to the in-depth analysis of the lived experience concerning the phenomena on which it is focused. Two, IPA is a method focused on how experiential phenomena are understood from a particular perspective (Drinkwater et al., 2013; Walsh et al., 2020).

Findings within this study are credible to the extent that three of the authors possessed expertise in qualitative analysis. Furthermore, the analytical process involved triangulation of researcher interpretations and acknowledgement of divergent narratives. Furthermore, participants were sampled from a range of backgrounds and levels of professed ability. This included paranormal practitioners (i.e., professional psychics and mediums) and laypersons with less formalised abilities. The interviewer possessed good knowledge of the paranormal, and hence, although a compassionate sceptic, was able to appreciate the content and nature of narratives. This ensured that interviewees were able to express themselves in an open manner.

## Data Availability Statement

The raw data supporting the conclusions of this article will be made available by the authors, without undue reservation.

## Ethics Statement

The studies involving human participants were reviewed and approved by Manchester Metropolitan University Faculty of Health, Psychology and Social Care Ethics Committee. The patients/participants provided their written informed consent to participate in this study.

## Author Contributions

KD and ND provided the theoretical focus, developed content, and produced the initial article. KD was responsible for data collection and initial analysis. LS, MP, and SW performed the RTA. AD reviewed the data analysis and wrote up the results. All authors were involved in the final proofs and subsequent submission of the article.

## Conflict of Interest

AD was employed by Adelphi Values Ltd. The remaining authors declare that the research was conducted in the absence of any commercial or financial relationships that could be construed as a potential conflict of interest.

## Publisher’s Note

All claims expressed in this article are solely those of the authors and do not necessarily represent those of their affiliated organizations, or those of the publisher, the editors and the reviewers. Any product that may be evaluated in this article, or claim that may be made by its manufacturer, is not guaranteed or endorsed by the publisher.

## References

[B1] Andrews T. (2012). What is social constructionism? *Ground. Theory Rev.* 11 39–46.

[B2] Ashworth P. (2008). “Conceptual foundations of qualitative psychology,” in *Qualitative psychology: A practical guide to research methods*, ed. Smith J. A. (London: Sage).

[B3] Ashworth P. D. (2015). The lifeworld – enriching qualitative evidence. *Qual. Res. Psychol.* 13 20–32. 10.1080/14780887.2015.1076917

[B4] Ayar D. Aksu C. Caki B. Gungormus Z. (2022). The Relationship Between Paranormal Beliefs, Social Efficacy and Social Outcome Expectations in Muslim Society: the Case of Turkey. *J. Rel. Health* 2022 1–18. 10.1007/s10943-021-01467-4 34978006 PMC8720552

[B5] Beischel J. (2007). Contemporary Methods used in Laboratory-Based Mediumship Research. *J. Parapsychol.* 71 37–68.

[B6] Beischel J. Mosher C. Boccuzzi M. (2017). Quantitative and qualitative analyses of mediumistic and psychic experiences. *Threshold: J. Interdiscipl. Consc. Stud.* 1 51–91.

[B7] Boyatzis R. E. (1998). *Transforming qualitative information: Thematic analysis and code development.* Thousand Oaks, CA: Sage.

[B8] Bragaru M. Van Wilgen C. P. Geertzen J. H. Ruijs S. G. Dijkstra P. U. Dekker R. (2013). Barriers and facilitators of participation in sports: a qualitative study on Dutch individuals with lower limb amputation. *PLoS One* 8:e59881. 10.1371/journal.pone.0059881 23533655 PMC3606215

[B9] Braun V. Clarke V. (2006). Using thematic analysis in psychology. *Q. Psychol.* 3 77–101. 10.1191/1478088706qp063oa 32100154

[B10] Braun V. Clarke V. (2012). “Thematic Analysis,” in *APA handbook of research methods in psychology*, eds Cooper H. M. Camic P. M. Long D. L. Panter A. T. Rindskopf D. Sher K. J. (Washington, D.C: American Psychological Association).

[B11] Braun V. Clarke V. (2013). *Successful qualitative research: A practical guide for beginners.* Thousand Oaks, CA: Sage.

[B12] Braun V. Clarke V. (2014). What can “thematic analysis” offer health and wellbeing researchers? *Internat. J. Q. Stud. Health Well-Being* 9:26152. 10.3402/qhw.v9.26152 25326092 PMC4201665

[B13] Braun V. Clarke V. (2019). Reflecting on reflexive thematic analysis. *Q. Res. Sport Exerc. Health* 11 589–597. 10.1080/2159676X.2019.1628806

[B14] Braun V. Clarke V. (2020). One size fits all? What counts as quality practice in (reflexive) thematic analysis? *Q. Res. Psychol.* 18 328–352. 10.1080/14780887.2020.1769238

[B15] Byrne D. (2021). A worked example of Braun and Clarke’s approach to reflexive thematic analysis. *Qual. Quant.* 2021 1–22. 10.1007/s11135-021-01182-y

[B16] Campbell K. A. Orr E. Durepos P. Nguyen L. Li L. Whitmore C. (2021). Reflexive thematic analysis for applied qualitative health research. *Q. Rep.* 26 2011–2028. 10.46743/2160-3715/2021.5010

[B17] Dagnall N. Drinkwater K. Denovan A. Parker A. (2015). Suggestion, belief in the paranormal, proneness to reality testing deficits and perception of an allegedly haunted building. *J. Parapsychol.* 79 87–104.

[B18] Dagnall N. Elliott C. Drinkwater K. Denovan A. Parker A. (2020). Predictors of hearing electronic voice phenomena in random noise: schizotypy, fantasy proneness, and paranormal beliefs. *J. Parapsychol.* 84 96–113. 10.30891/jopar.2020.01.09

[B19] Dagnall N. Parker A. Munley G. Drinkwater K. (2010). Common paranormal belief dimensions. *J. Sci. Expl.* 24 431–477.

[B20] Dagnall N. A. Drinkwater K. Parker A. Clough P. (2016). Paranormal experience, belief in the paranormal and anomalous beliefs. *Paranthropology* 7 4–15.

[B21] Damasio A. (2018). *The strange order of things: Life, feeling, and the making of cultures.* New York, NY: Knopf Doubleday Publishing Group.

[B22] Damasio A. R. (1999). *The feeling of what happens: Body and emotion in the making of consciousness.* Boston: Houghton Mifflin Harcourt.

[B23] Daniels M. (2005). *Shadow, self, spirit: Essays in transpersonal psychology.* Exeter, UK: Imprint Academic.

[B24] Dattel A. R. Neimeyer R. A. (1990). Sex differences in death anxiety: Testing the emotional expressiveness hypothesis. *Death Stud.* 14 1–11. 10.1080/07481189008252341

[B25] Denovan A. Dagnall N. Drinkwater K. Parker A. (2018). Latent profile analysis of schizotypy and paranormal belief: associations with probabilistic reasoning performance. *Front. Psychol.* 9:35. 10.3389/fpsyg.2018.00035 29434562 PMC5791384

[B26] Drinkwater K. Dagnall N. Bate L. (2013). Into the unknown: using interpretative phenomenological analysis to explore personal accounts of paranormal experiences. *J. Parapsychol.* 77 281–294.

[B27] Drinkwater K. Dagnall N. Grogan S. Riley V. (2017a). Understanding the unknown: A thematic analysis of subjective paranormal experiences. *Austr. J. Parapsychol.* 17 23–46.

[B28] Drinkwater K. Denovan A. Dagnall N. Parker A. (2017b). An assessment of the dimensionality and factorial structure of the revised paranormal belief scale. *Front. Psychol.* 8:1693. 10.3389/fpsyg.2017.01693 29018398 PMC5622942

[B29] Drinkwater K. Denovan A. Dagnall N. Parker A. (2018). The Australian sheep-goat scale: an evaluation of factor structure and convergent validity. *Front. Psychol.* 9:1594. 10.3389/fpsyg.2018.01594 30210415 PMC6121071

[B30] Drinkwater K. Laythe B. Houran J. Dagnall N. O’Keeffe C. Hill S. A. (2019). Exploring gaslighting effects via the VAPUS model for ghost narratives. *Austr. J. Parapsychol.* 19 143–179.

[B31] Drinkwater K. G. Denovan A. Dagnall N. (2020). Lucid dreaming, nightmares, and sleep paralysis: associations with reality testing deficits and paranormal experience/belief. *Front. Psychol.* 11:471. 10.3389/fpsyg.2020.00471 32256437 PMC7093643

[B32] Drinkwater K. G. Dagnall N. Denovan A. Williams C. (2021a). Differences in cognitive-perceptual factors arising from variations in self-professed paranormal ability. *Front. Psychol.* 12:2258. 10.3389/fpsyg.2021.681520 34177736 PMC8222626

[B33] Drinkwater K. G. Dagnall N. Denovan A. Williams C. (2021b). Paranormal belief, thinking style and delusion formation: a latent profile analysis of within-individual variations in experience-based paranormal facets. *Front. Psychol.* 12:670959. 10.3389/fpsyg.2021.670959 34262510 PMC8273333

[B34] Drinkwater K. G. Dagnall N. Denovan A. Parker A. Álex Escolà-Gascón. (2021c). Executive functioning: assessing the role of perceived paranormal ability. *Front. Psychol.* 12:79828. 10.3389/fpsyg.2021.798283 35002892 PMC8733669

[B35] Drinkwater K. G. Dagnall N. Denovan A. Parker A. Álex Escolà-Gascón. (2022). Paranormal experience profiles and their association with variations in executive functions: A latent profile analysis. *Front. Psychol.* 12:778312. 10.3389/fpsyg.2021.778312 35082722 PMC8784801

[B36] D’Souza A. Fabricius A. Amodio V. Colquhoun H. Lewko J. Haag H. (2020). Men’s gendered experiences of rehabilitation and recovery following traumatic brain injury: a reflexive thematic analysis. *Neuropsychol. Rehabilit.* 2020 1–22. 10.1080/09602011.2020.1822882 32960149

[B37] Gibson B. Umeh K. Davies I. Newson L. (2021). The best possible self-intervention as a viable public health tool for the prevention of type 2 diabetes: a reflexive thematic analysis of public experience and engagement. *Health Expect.* 24 1713–1724. 10.1111/hex.13311 34258837 PMC8483206

[B38] Göritz A. S. Schumacher J. (2000). The WWW as a research medium: An illustrative survey on paranormal belief. *Percept. Motor Skills* 90 1195–1206. 10.2466/pms.2000.90.3c.1195 10939070

[B39] Grof S. (1975). *Realms of the human unconscious: Observations from LSD research.* New York, NY: The Viking Press.

[B40] Guest G. Bunce A. Johnson L. (2006). How many interviews are enough? An experiment with data saturation and variability. *Field Methods* 18 59–82. 10.1177/1525822X05279903

[B41] Irwin H. J. (2009). *The psychology of paranormal belief: A researcher’s handbook.* Hatfield AL: University of Hertfordshire Press.

[B42] Irwin H. J. Dagnall N. Drinkwater K. (2013). Parapsychological experience as anomalous experience plus paranormal attribution: a questionnaire based on a new approach to measurement. *J. Parapsychol.* 77 39–53. 10.1037/t31377-000

[B43] Kuzel A. (1999). “Sampling in qualitative inquiry,” in *Doing qualitative research*, 2nd Edn, eds Miller W. Crabtree B. (Thousand Oaks, CA: Sage), 33–45.

[B44] Kuzel A. J. (1992). “Sampling in qualitative inquiry,” in *Doing Qualitative Research*, eds Crabtree B. F. Miller W. L. (Newbury Park, CA: Sage), 31–44.

[B45] Lange R. Ross R. M. Dagnall N. Irwin H. J. Houran J. Drinkwater K. (2019). Anomalous experiences and paranormal attributions: psychometric challenges in studying their measurement and relationship. *Psychol. Consc.* 6 346–358. 10.1037/cns0000187

[B46] Laythe B. Houran J. Dagnall N. Drinkwater K. (2021). Conceptual and clinical implications of a “Haunted People Syndrome”. *Spiritual. Clin. Pract.* 8 195–214. 10.1037/scp0000251

[B47] Liu N. Nikitas A. Parkinson S. (2020). Exploring expert perceptions about the cyber security and privacy of Connected and Autonomous Vehicles: a thematic analysis approach. *Transport. Res. Part F* 75 66–86. 10.1016/j.trf.2020.09.019

[B48] Lobato E. Mendoza J. Sims V. Chin M. (2014). Examining the relationship between conspiracy theories, paranormal beliefs, and pseudoscience acceptance among a university population. *Appl. Cogn. Psychol.* 28 617–625. 10.1002/acp.3042

[B49] McGrew J. H. McFall R. M. (1990). A scientific inquiry into the validity of astrology. *J. Sci. Expl.* 4 75–83.

[B50] Morse J. M. (1994). “Designing funded qualitative research,” in *Handbook of Qualitative Research*, eds Denzin N. K. Lincoln Y. S. (Thousand Oaks, CA: Sage), 220–235.

[B51] Murray C. sD. Wooffitt R. (2010). Anomalous experience and qualitative research: An introduction to the special issue. *Q. Res. Psychol.* 7 1–4. 10.1080/14780880903304535

[B52] O’Keeffe C. Wiseman R. (2005). Testing alleged mediumship: Methods and results. *Br. J. Psychol.* 96 165–179. 10.1348/000712605X36361 15969829

[B53] O’Keeffe C. Houran J. Houran D. J. Dagnall N. Drinkwater K. Sheridan L. (2019). The Dr. John Hall story: a case study in putative “Haunted People Syndrome”. *Ment. Health Relig. Cult*. 22, 910–929. 10.1080/13674676.2019.1674795

[B54] Osty E. (1913). *Lucidité et Intuition: étude Expérimentale.* London: Forgotten Books.

[B55] Osty E. (1926). *Pascal Forthuny: Une Faculté de Connaissance Supranormale.* Paris: Félix Alcan.

[B56] Parker R. K. B. (2018). “*Phenomenology of Mysticism*, Introduction and Chapter One,” in *Gerda Walther’s Phenomenology of Sociality, Psychology, and Religion. Women in the History of Philosophy and Sciences*, Vol. 2 ed. Calcagno A. (Cham: Springer). 10.1007/978-3-319-97592-4_9

[B57] Parra A. Argibay J. C. (2009). An Experimental Study with Ordinary People for Testing ‘Sacred’ Objects through Psi Detection. *J. Soc. Psych. Res.* 73 40–48.

[B58] Roberson Q. Perry J. L. (2021). Inclusive Leadership in Thought and Action: A Thematic Analysis. *Group Org. Manag.* 2021:10596011211013161. 10.1177/10596011211013161

[B59] Rock A. J. Beischel J. Cott C. C. (2009). Psi vs. survival: a qualitative investigation of mediums’ phenomenology comparing psychic readings and ostensible communication with the deceased. *Transp. Psychol. Rev.* 13 76–89.

[B60] Rock A. J. Beischel J. Schwartz G. E. (2008). Thematic analysis of research mediums’ experiences of discarnate communication. *J. Sci. Expl.* 22 179–192.

[B61] Roxburgh E. C. Roe C. A. (2011). A survey of dissociation, boundary-thinness, and psychological wellbeing in spiritualist mental mediumship. *J. Parapsychol.* 75 279–299.

[B62] Roxburgh E. C. Roe C. A. (2013). “Say from whence you owe this strange intelligence”: investigating explanatory systems of spiritualist mental mediumship using interpretative phenomenological analysis. *Internat. J. Transp. Stud.* 32 27–42. 10.24972/ijts.2013.32.1.27

[B63] Schmied-Knittel I. Schetsche M. T. (2005). Everyday miracles: results of a representative survey in Germany. *Eur. J. Parapsychol.* 20 3–21.

[B64] Smith J. A. Flowers P. Larkin M. (2022). *Interpretative Phenomenological Analysis.* Thousand Oaks: Sage Publications, 10.1037/0000259-001

[B65] Terry G. Hayfield N. Clarke V. Braun V. (2017). “Thematic analysis,” in *The SAGE Handbook of Qualitative Research in Psychology*, eds Willig C. Rogers W. (London: SAGE Publications Ltd), 17–36. 10.4135/9781526405555

[B66] The British Psychological Society (2021). *BPS Code of Human Research Ethics.* Available online at: https://www.bps.org.uk/sites/www.bps.org.uk/files/Policy/Policy%20-%20Files/BPS%20Code%20of%20Human%20Research%20Ethics.pdf (accessed date April 2021)

[B67] Vaismoradi M. Turunen H. Bondas T. (2013). Content analysis and thematic analysis: Implications for conducting a qualitative descriptive study. *Nurs. Sci.* 15 398–405. 10.1111/nhs.12048 23480423

[B68] Walsh R. S. Crawley L. Dagnall N. Fortune D. G. (2020). The man who used to shrug – one man’s lived experience of TBI. *NeuroRehabilitation* 47 11–24. 10.3233/nre-203079 32675423

[B69] Wilde D. Murray C. D. (2010). Interpreting the anomalous: Finding meaning in out-of-body and near-death experiences. *Q. Psychol.* 7 57–72.

[B70] Williams L. M. Irwin H. J. (1991). A study of paranormal belief, magical ideation as an index of schizotypy and cognitive style. *Personal. Indiv. Diff.* 12 1339–1348. 10.1016/0191-8869(91)90210-3

[B71] Willig C. (2013). *Introducing Qualitative Research in Psychology.* New York, NY: McGraw-Hill Education.

